# *catena*-Poly[[[bis­(acetato-κ^2^ *O*,*O*′)aqua­cadmium]-μ-[(pyridin-3-yl)(pyridin-4-yl)methanone]-κ^2^ *N*:*N*′] dihydrate]

**DOI:** 10.1107/S1600536812019101

**Published:** 2012-05-05

**Authors:** Zhi-Wei Wang, Ai-Min Li, Ya Zhang

**Affiliations:** a Department of Chemistry, Capital Normal University, Beijing 100048, People’s Republic of China

## Abstract

In the title complex, {[Cd(CH_3_COO)_2_(C_11_H_8_N_2_O)(H_2_O)]·2H_2_O}_*n*_, the Cd^II^ ion adopts an O_5_N_2_ penta­gonal–bipyramidal coordination geometry with four acetate O atoms and one water O atom at the equatorial sites and two pyridine N atoms at the axial sites. The (pyridin-3-yl)(pyridin-4-yl)methanone ligand acts in a μ_2_-bridging mode, linking the metal atoms, leading to an infinite chain along [-110]. O—H⋯O hydrogen bonds involving the lattice water mol­ecules connect these chains into a three-dimensional network.

## Related literature

For the coordination chemistry of pyridyl-based derivatives, see: Zhao *et al.* (2004 ▶); Wang *et al.* (2009 ▶). For background to di-2-pyridinyl­methanone see: Boudalis *et al.* (2003 ▶). For the transition metal complexes of the positional isomers of di-2-pyridinyl­methanone, see: Chen, Guo *et al.* (2005 ▶); Chen *et al.* (2009 ▶); Chen, Du & Mak (2005 ▶); Chen & Mak (2005 ▶); Famum *et al.* (2009 ▶).
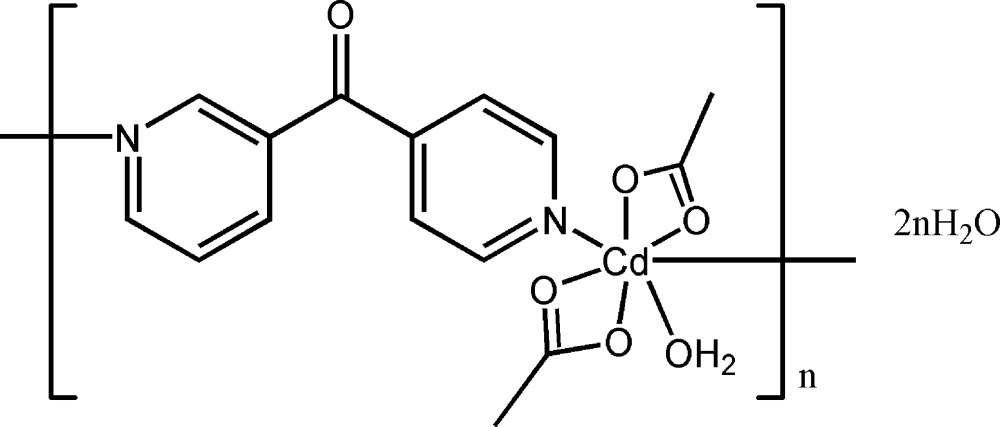


## Experimental

### 

#### Crystal data

[Cd(C_2_H_3_O_2_)_2_(C_11_H_8_N_2_O)(H_2_O)]·2H_2_O
*M* *_r_* = 468.73
Triclinic, 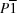

*a* = 8.545 (2) Å
*b* = 10.699 (3) Å
*c* = 11.215 (3) Å
α = 76.903 (5)°
β = 87.833 (5)°
γ = 77.160 (5)°
*V* = 973.5 (5) Å^3^
*Z* = 2
Mo *K*α radiation
μ = 1.16 mm^−1^
*T* = 293 K
0.38 × 0.20 × 0.18 mm

#### Data collection

Bruker SMART APEXII CCD area-detector diffractometer
Absorption correction: multi-scan (*SADABS*; Bruker, 2007 ▶) *T*_min_ = 0.840, *T*_max_ = 1.000
6828 measured reflections
4747 independent reflections
4041 reflections with *I* > 2σ(*I*)
*R*_int_ = 0.023

#### Refinement

*R*[*F* ^2^ > 2σ(*F* ^2^)] = 0.033
*wR*(*F* ^2^) = 0.084
*S* = 1.05
4747 reflections
235 parameters
H-atom parameters constrained
Δρ_max_ = 0.55 e Å^−3^
Δρ_min_ = −0.33 e Å^−3^

### 

Data collection: *APEX2* (Bruker, 2007 ▶); cell refinement: *APEX2* and *SAINT* (Bruker, 2007 ▶); data reduction: *SAINT*; program(s) used to solve structure: *SHELXS97* (Sheldrick, 2008 ▶); program(s) used to refine structure: *SHELXL97* (Sheldrick, 2008 ▶); molecular graphics: *SHELXTL* (Sheldrick, 2008 ▶); software used to prepare material for publication: *SHELXTL* and *PLATON* (Spek, 2009 ▶).

## Supplementary Material

Crystal structure: contains datablock(s) I, global. DOI: 10.1107/S1600536812019101/bt5902sup1.cif

Structure factors: contains datablock(s) I. DOI: 10.1107/S1600536812019101/bt5902Isup2.hkl

Additional supplementary materials:  crystallographic information; 3D view; checkCIF report

## Figures and Tables

**Table 1:** Hydrogen-bond geometry (Å, °)

*D*—H⋯*A*	*D*—H	H⋯*A*	*D*⋯*A*	*D*—H⋯*A*
O1*W*—H1*WA*⋯O4	0.89	2.06	2.773 (2)	136
O1*W*—H1*WB*⋯O2w	0.89	2.00	2.834 (3)	155
O2*W*—H2*WA*⋯O2	0.89	1.93	2.811 (4)	173
O2*W*—H2*WB*⋯O1w^i^	0.89	1.95	2.803 (2)	162
O3*W*—H3*WA*⋯O5^ii^	0.89	1.80	2.679 (2)	172
O3*W*—H3*WB*⋯O3^iii^	0.89	1.81	2.693 (3)	170

Symmetry codes: (i) 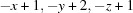; (ii) 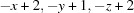; (iii) 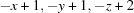.

**Fig. 1.**
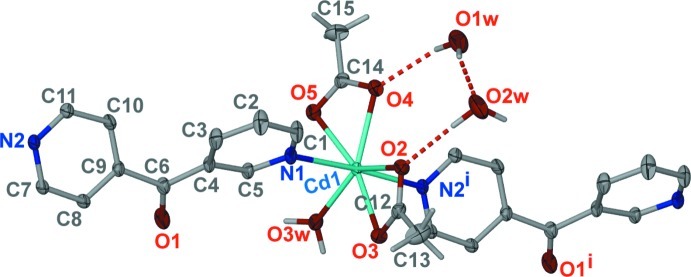
The title complex showing the atom-numbering scheme, with displacement ellipsoids shown at the 30% probability level. All aryl hydrogen atoms are omitted for clarity. Symmetry codes: (i) x - 1, y + 1, z.

**Fig. 2.**
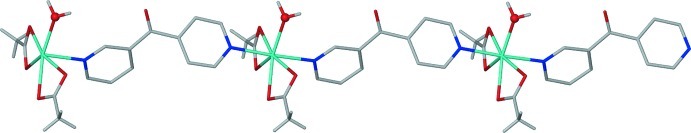
Infinite chain structure along the [-1 1 0] direction of the title complex.The O atoms of the water were shown as red balls, and all aryl H atoms are omitted for clarity.

**Fig. 3.**
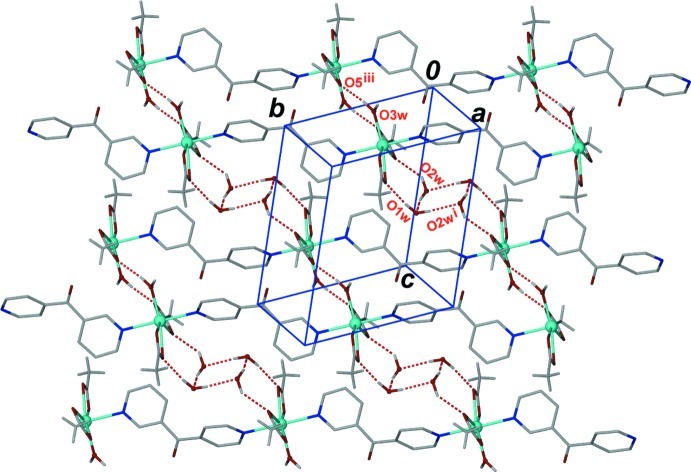
The hydrogen-bonding interactions that assemble with the infinite chain structures. The red-dashed lines represent hydrogen-bonding interactions. All water O atoms were shown as red balls, and all aryl H atoms are omitted for clarity.

## supplementary crystallographic information

### Comment

Pyridyl-based ligand are widely and successfully used to construction intriguing supramolecular archtectures with various transition metal salts (Zhao *et al.* 2004; Wang *et al.*, 2009). The coordination chemistry of di-2-pyridinylmethanone (di-2-pyridyl ketone, DPK), (2-C_5_H_4_N)_2_CO, has been phenomenally developed in the past decades (Boudalis *et al.*, 2003). The coordination chemistry of its positional isomers such di-3-pyridinylmethanone(Chen, Guo *et al.* 2005; Chen *et al.*, 2009),2-pyridinyl-3-pyridinylmethanone (Chen, Du & Mak, 2005) and 2-pyridinyl-4-pyridinylmethanone (Chen & Mak, 2005) have also be well explored. Herein, we report a new structure derived from 3-pyridinyl-4-pyridinylmethanone (Scheme 1), namely {[Cd(C_11_H_8_N_2_O)(CH_3_CO_2_)_2_(H_2_O)].2H_2_O}_∞_.

As shown in Fig. 1, the Cd^II^ ion adopts an O_5_N_2_-pentagonal bipyramid coordination geometry with four acetate O atoms and one auqa O3w atom at the equatorial sites and two pyridyl N atoms at the axial sites (Fig. 1). As shown in Fig. 2, The 3-pyridinyl-4-pyridinylmethanone (3,4'-dipyridyl ketone) ligand fonctions as a µ^2^-bridging mode linking to the Cd^II^ centers, leading to an infinite chain structure alone the [-110] direction. Such a 1-D structure sharply differs from the 2-D net of catena-[[bis(u_2_-3,4'-dipyridyl ketone-κ N:N')-diaqua-cadmium(II)] diperchlorate dihydrate] (Famum *et al.*, 2009) with 3-pyridinyl-4-pyridinylmethanone.Two interconnected lattice water molecules (O1w and O2w) respectively anchor to two separated acetate around the Cd^II^ center through hydrogen bonding interactions (Fig.1, Table 1), which further link to their symmetry-related ones to form a tetramer (O1w···O2w···O1w^ii^···O2w^ii^, see Fig. 3 and Table 1, symmetry code: ii -x + 1, -y + 2, -z + 1). The tetrameric water cluster with a invert center thus functions as a linkage to bridge the parallel chains together through H-bonding interactions, which combine the O3W—H3WB···O5^iii^(acetate) interactions to assemble with the infinite chains into a layer, as shown in Fig. 3 (iii -x + 2, -y + 1, -z + 2). Along the [110] direction, the almost parallel layers formed are stacked and stablized through another set of H-bonding interactions of O3w [O3W—H3WB···O3^iv^(acetate), iv -x+1, -y+1, -z+2. see Table 1], forming a three-dimensional frameworks.

### Experimental

The ligand was prepared according to the procedure of the literature reported (Chen & Mak 2005). The Cd(CH_3_CO_2_)_2_.2H_2_O (30 mg, 0.12 mmol) and 3-pyridinyl-4-pyridinylmethanone (19 mg, 0.1 mmol) were dissolved in a mixed solvent of 1 ml deionized water and 3 ml acetonitrile with stirring at room temperature. After 3 hours, the resulted clear solution was filtered and the filtrate was left to stand in air. The clolorless crystals suitable for x-ray diffraction analysis were deposited after about two weaks (23.9 mg, 52% yield).

### Refinement

All the H atoms were located in the difference electron density maps but were placed in idealized positions and allowed to ride on the carrier atoms, with O—H = 0.89 Å, C—H = 0.93 Å and 0.96 Å for aryl H and methyl H aotms, respectively, and *U*_iso_(H) = 1.2*U*_eq_(C) or *U*_iso_(H) = 1.5*U*_eq_(C_methyl_, O).

### Crystal data

**Table:**

[Cd(C_2_H_3_O_2_)_2_(C_11_H_8_N_2_O)(H_2_O)]·2H_2_O	*Z* = 2
*M**_r_* = 468.73	*F*(000) = 472
Triclinic, *P*1	*D*_x_ = 1.599 Mg m^−^^3^
Hall symbol: -P 1	Mo *K*α radiation, λ = 0.71073 Å
*a* = 8.545 (2) Å	Cell parameters from 215 reflections
*b* = 10.699 (3) Å	θ = 1.9–28.4°
*c* = 11.215 (3) Å	µ = 1.16 mm^−^^1^
α = 76.903 (5)°	*T* = 293 K
β = 87.833 (5)°	Block, colorless
γ = 77.160 (5)°	0.38 × 0.20 × 0.18 mm
*V* = 973.5 (5) Å^3^	

### Data collection

**Table:**

Bruker SMART APEXII CCD area-detector diffractometer	4747 independent reflections
Radiation source: fine-focus sealed tube	4041 reflections with *I* > 2σ(*I*)
Graphite monochromator	*R*_int_ = 0.023
ω scans	θ_max_ = 28.4°, θ_min_ = 1.9°
Absorption correction: multi-scan (*SADABS*; Bruker, 2007)	*h* = −11→11
*T*_min_ = 0.840, *T*_max_ = 1.000	*k* = −9→14
6828 measured reflections	*l* = −14→14

### Refinement

**Table:**

Refinement on *F*^2^	Primary atom site location: structure-invariant direct methods
Least-squares matrix: full	Secondary atom site location: difference Fourier map
*R*[*F*^2^ > 2σ(*F*^2^)] = 0.033	Hydrogen site location: inferred from neighbouring sites
*wR*(*F*^2^) = 0.084	H-atom parameters constrained
*S* = 1.05	*w* = 1/[σ^2^(*F*_o_^2^) + (0.0412*P*)^2^] *P* = (*F*_o_^2^ + 2*F*_c_^2^)/3
4747 reflections	(Δ/σ)_max_ = 0.001
235 parameters	Δρ_max_ = 0.55 e Å^−^^3^
0 restraints	Δρ_min_ = −0.33 e Å^−^^3^

### Special details

**Table:**

Geometry. All esds (except the esd in the dihedral angle between two l.s. planes) are estimated using the full covariance matrix. The cell esds are taken into account individually in the estimation of esds in distances, angles and torsion angles; correlations between esds in cell parameters are only used when they are defined by crystal symmetry. An approximate (isotropic) treatment of cell esds is used for estimating esds involving l.s. planes.
Refinement. Refinement of F^2^ against ALL reflections. The weighted R-factor wR and goodness of fit S are based on F^2^, conventional R-factors R are based on F, with F set to zero for negative F^2^. The threshold expression of F^2^ > 2sigma(F^2^) is used only for calculating R-factors(gt) etc. and is not relevant to the choice of reflections for refinement. R-factors based on F^2^ are statistically about twice as large as those based on F, and R- factors based on ALL data will be even larger.

### Fractional atomic coordinates and isotropic or equivalent isotropic displacement parameters (Å^2^)

**Table:**

	*x*	*y*	*z*	*U*_iso_*/*U*_eq_	
Cd1	0.73166 (2)	0.608007 (18)	0.821278 (16)	0.03377 (8)	
N1	0.8989 (3)	0.4149 (2)	0.7726 (2)	0.0405 (6)	
N2	1.5508 (3)	−0.2090 (2)	0.8722 (2)	0.0417 (6)	
O1	1.1137 (3)	0.0944 (3)	1.0377 (2)	0.0685 (8)	
C1	0.9091 (4)	0.4024 (4)	0.6568 (3)	0.0582 (10)	
H1A	0.8491	0.4689	0.5971	0.070*	
C2	1.0037 (5)	0.2965 (4)	0.6219 (3)	0.0688 (12)	
H2A	1.0097	0.2928	0.5398	0.083*	
C3	1.0909 (4)	0.1944 (3)	0.7093 (3)	0.0548 (9)	
H3A	1.1541	0.1205	0.6875	0.066*	
C4	1.0812 (3)	0.2054 (3)	0.8303 (3)	0.0391 (6)	
C5	0.9845 (3)	0.3188 (3)	0.8561 (3)	0.0384 (6)	
H5A	0.9798	0.3276	0.9369	0.046*	
C6	1.1627 (4)	0.0998 (3)	0.9342 (3)	0.0435 (7)	
C7	1.4426 (4)	−0.2286 (3)	0.9592 (3)	0.0463 (7)	
H7A	1.4505	−0.3132	1.0067	0.056*	
C8	1.3195 (4)	−0.1299 (3)	0.9822 (3)	0.0448 (7)	
H8A	1.2480	−0.1481	1.0450	0.054*	
C9	1.3027 (3)	−0.0036 (3)	0.9114 (2)	0.0380 (6)	
C10	1.4176 (4)	0.0188 (3)	0.8233 (3)	0.0462 (7)	
H10A	1.4137	0.1028	0.7757	0.055*	
C11	1.5389 (4)	−0.0866 (3)	0.8074 (3)	0.0464 (7)	
H11A	1.6157	−0.0706	0.7483	0.056*	
O5	0.9921 (3)	0.6479 (2)	0.85165 (19)	0.0476 (5)	
O2	0.5727 (3)	0.6074 (2)	0.64724 (19)	0.0518 (6)	
O3	0.5016 (3)	0.5111 (2)	0.82736 (19)	0.0484 (5)	
O4	0.8583 (3)	0.7585 (2)	0.68529 (19)	0.0520 (5)	
C14	0.9794 (4)	0.7337 (3)	0.7540 (3)	0.0421 (7)	
C15	1.1079 (5)	0.8105 (4)	0.7191 (4)	0.0762 (12)	
H15A	1.1939	0.7607	0.6802	0.114*	
H15B	1.1481	0.8276	0.7912	0.114*	
H15C	1.0637	0.8924	0.6635	0.114*	
C12	0.4849 (4)	0.5416 (3)	0.7123 (3)	0.0453 (7)	
C13	0.3582 (5)	0.4955 (5)	0.6545 (4)	0.0817 (13)	
H13A	0.3583	0.5274	0.5673	0.122*	
H13B	0.2548	0.5287	0.6856	0.122*	
H13C	0.3807	0.4011	0.6737	0.122*	
O3W	0.7517 (3)	0.5164 (3)	1.02658 (19)	0.0601 (7)	
O1W	0.7280 (4)	0.9615 (3)	0.4905 (3)	0.0903 (10)	
O2W	0.4924 (4)	0.8260 (3)	0.4497 (3)	0.0989 (11)	
H2WA	0.5192	0.7528	0.5080	0.148*	
H2WB	0.4068	0.8825	0.4682	0.148*	
H1WB	0.6774	0.9079	0.4650	0.148*	
H3WA	0.8372	0.4677	1.0709	0.148*	
H3WB	0.6751	0.5070	1.0818	0.148*	
H1WA	0.7817	0.9348	0.5615	0.148*	

### Atomic displacement parameters (Å^2^)

**Table:**

	*U*^11^	*U*^22^	*U*^33^	*U*^12^	*U*^13^	*U*^23^
Cd1	0.03121 (12)	0.02824 (11)	0.03400 (12)	0.00510 (7)	0.00321 (7)	−0.00279 (8)
N1	0.0415 (13)	0.0352 (13)	0.0381 (12)	0.0071 (10)	−0.0005 (10)	−0.0097 (10)
N2	0.0393 (13)	0.0308 (12)	0.0453 (13)	0.0059 (10)	0.0064 (10)	−0.0027 (10)
O1	0.0837 (19)	0.0541 (15)	0.0451 (12)	0.0237 (13)	0.0146 (12)	−0.0053 (11)
C1	0.062 (2)	0.054 (2)	0.0431 (17)	0.0222 (17)	−0.0081 (15)	−0.0130 (15)
C2	0.079 (3)	0.073 (3)	0.0398 (17)	0.029 (2)	−0.0093 (16)	−0.0260 (17)
C3	0.058 (2)	0.0506 (19)	0.0475 (17)	0.0171 (16)	−0.0052 (15)	−0.0219 (15)
C4	0.0375 (15)	0.0319 (14)	0.0416 (15)	0.0074 (11)	−0.0004 (11)	−0.0103 (12)
C5	0.0388 (15)	0.0332 (15)	0.0380 (14)	0.0055 (12)	0.0028 (11)	−0.0109 (12)
C6	0.0488 (17)	0.0332 (15)	0.0428 (15)	0.0041 (13)	0.0027 (13)	−0.0103 (12)
C7	0.0456 (17)	0.0302 (15)	0.0507 (17)	0.0053 (12)	0.0112 (13)	0.0012 (13)
C8	0.0457 (17)	0.0333 (15)	0.0453 (16)	0.0015 (13)	0.0140 (13)	−0.0002 (13)
C9	0.0393 (15)	0.0320 (14)	0.0362 (14)	0.0058 (11)	0.0024 (11)	−0.0075 (11)
C10	0.0501 (18)	0.0297 (15)	0.0476 (16)	0.0032 (13)	0.0068 (13)	0.0012 (13)
C11	0.0425 (17)	0.0376 (16)	0.0496 (17)	0.0018 (13)	0.0105 (13)	−0.0022 (13)
O5	0.0453 (12)	0.0503 (13)	0.0417 (11)	−0.0059 (10)	−0.0038 (9)	−0.0024 (10)
O2	0.0492 (13)	0.0550 (14)	0.0410 (11)	−0.0053 (11)	0.0027 (9)	0.0038 (10)
O3	0.0477 (12)	0.0551 (14)	0.0379 (11)	−0.0097 (10)	0.0043 (9)	−0.0030 (10)
O4	0.0488 (13)	0.0518 (13)	0.0474 (12)	−0.0076 (10)	−0.0047 (10)	0.0027 (10)
C14	0.0388 (16)	0.0416 (16)	0.0456 (16)	−0.0042 (13)	0.0069 (12)	−0.0149 (14)
C15	0.071 (3)	0.080 (3)	0.085 (3)	−0.039 (2)	0.014 (2)	−0.015 (2)
C12	0.0355 (15)	0.0491 (18)	0.0477 (17)	0.0001 (13)	0.0037 (13)	−0.0129 (14)
C13	0.069 (3)	0.124 (4)	0.070 (3)	−0.035 (3)	0.007 (2)	−0.046 (3)
O3W	0.0433 (12)	0.0789 (18)	0.0368 (11)	0.0075 (12)	0.0039 (9)	0.0095 (11)
O1W	0.110 (3)	0.079 (2)	0.0571 (16)	0.0117 (18)	0.0076 (16)	0.0015 (15)
O2W	0.129 (3)	0.067 (2)	0.0707 (19)	0.0134 (19)	0.0083 (18)	0.0106 (16)

### Geometric parameters (Å, º)

**Table:**

Cd1—O3W	2.286 (2)	C7—H7A	0.9300
Cd1—O4	2.372 (2)	C8—C9	1.383 (4)
Cd1—N2^i^	2.375 (2)	C8—H8A	0.9300
Cd1—N1	2.398 (2)	C9—C10	1.386 (4)
Cd1—O5	2.408 (2)	C10—C11	1.391 (4)
Cd1—O3	2.410 (2)	C10—H10A	0.9300
Cd1—O2	2.422 (2)	C11—H11A	0.9300
Cd1—C14	2.747 (3)	O5—C14	1.250 (4)
N1—C5	1.323 (3)	O2—C12	1.245 (4)
N1—C1	1.333 (4)	O3—C12	1.262 (4)
N2—C11	1.330 (4)	O4—C14	1.257 (4)
N2—C7	1.332 (4)	C14—C15	1.502 (5)
N2—Cd1^ii^	2.375 (2)	C15—H15A	0.9600
O1—C6	1.213 (4)	C15—H15B	0.9600
C1—C2	1.364 (5)	C15—H15C	0.9600
C1—H1A	0.9300	C12—C13	1.507 (5)
C2—C3	1.384 (4)	C13—H13A	0.9600
C2—H2A	0.9300	C13—H13B	0.9600
C3—C4	1.386 (4)	C13—H13C	0.9600
C3—H3A	0.9300	O3W—H3WA	0.8900
C4—C5	1.391 (4)	O3W—H3WB	0.8900
C4—C6	1.494 (4)	O1W—H1WB	0.8902
C5—H5A	0.9300	O1W—H1WA	0.8899
C6—C9	1.496 (4)	O2W—H2WA	0.8900
C7—C8	1.378 (4)	O2W—H2WB	0.8900
			
O3W—Cd1—O4	134.73 (9)	O1—C6—C4	120.6 (3)
O3W—Cd1—N2^i^	86.76 (9)	O1—C6—C9	118.9 (3)
O4—Cd1—N2^i^	88.26 (9)	C4—C6—C9	120.5 (2)
O3W—Cd1—N1	92.22 (8)	N2—C7—C8	123.3 (3)
O4—Cd1—N1	95.29 (9)	N2—C7—H7A	118.3
N2^i^—Cd1—N1	175.86 (9)	C8—C7—H7A	118.3
O3W—Cd1—O5	83.51 (8)	C7—C8—C9	119.7 (3)
O4—Cd1—O5	54.23 (7)	C7—C8—H8A	120.2
N2^i^—Cd1—O5	103.67 (9)	C9—C8—H8A	120.2
N1—Cd1—O5	80.18 (9)	C8—C9—C10	117.5 (3)
O3W—Cd1—O3	84.81 (8)	C8—C9—C6	118.4 (3)
O4—Cd1—O3	139.62 (7)	C10—C9—C6	124.2 (3)
N2^i^—Cd1—O3	86.02 (9)	C9—C10—C11	118.9 (3)
N1—Cd1—O3	89.90 (9)	C9—C10—H10A	120.6
O5—Cd1—O3	164.34 (7)	C11—C10—H10A	120.6
O3W—Cd1—O2	138.18 (9)	N2—C11—C10	123.5 (3)
O4—Cd1—O2	87.04 (8)	N2—C11—H11A	118.3
N2^i^—Cd1—O2	93.99 (9)	C10—C11—H11A	118.3
N1—Cd1—O2	84.09 (8)	C14—O5—Cd1	91.75 (18)
O5—Cd1—O2	136.00 (7)	C12—O2—Cd1	92.67 (19)
O3—Cd1—O2	53.64 (7)	C12—O3—Cd1	92.80 (19)
O3W—Cd1—C14	109.10 (9)	C14—O4—Cd1	93.23 (18)
O4—Cd1—C14	27.19 (8)	O5—C14—O4	120.7 (3)
N2^i^—Cd1—C14	96.00 (9)	O5—C14—C15	120.0 (3)
N1—Cd1—C14	88.13 (9)	O4—C14—C15	119.3 (3)
O5—Cd1—C14	27.06 (8)	O5—C14—Cd1	61.19 (16)
O3—Cd1—C14	166.01 (8)	O4—C14—Cd1	59.58 (16)
O2—Cd1—C14	112.38 (8)	C15—C14—Cd1	176.2 (2)
C5—N1—C1	117.5 (2)	C14—C15—H15A	109.5
C5—N1—Cd1	122.83 (18)	C14—C15—H15B	109.5
C1—N1—Cd1	119.61 (19)	H15A—C15—H15B	109.5
C11—N2—C7	117.1 (2)	C14—C15—H15C	109.5
C11—N2—Cd1^ii^	122.53 (19)	H15A—C15—H15C	109.5
C7—N2—Cd1^ii^	119.83 (19)	H15B—C15—H15C	109.5
N1—C1—C2	122.9 (3)	O2—C12—O3	120.8 (3)
N1—C1—H1A	118.5	O2—C12—C13	120.3 (3)
C2—C1—H1A	118.5	O3—C12—C13	118.9 (3)
C1—C2—C3	119.8 (3)	C12—C13—H13A	109.5
C1—C2—H2A	120.1	C12—C13—H13B	109.5
C3—C2—H2A	120.1	H13A—C13—H13B	109.5
C2—C3—C4	118.1 (3)	C12—C13—H13C	109.5
C2—C3—H3A	120.9	H13A—C13—H13C	109.5
C4—C3—H3A	120.9	H13B—C13—H13C	109.5
C3—C4—C5	117.8 (3)	Cd1—O3W—H3WA	129.2
C3—C4—C6	123.5 (3)	Cd1—O3W—H3WB	130.0
C5—C4—C6	118.6 (2)	H3WA—O3W—H3WB	100.0
N1—C5—C4	123.9 (3)	H1WB—O1W—H1WA	120.0
N1—C5—H5A	118.1	H2WA—O2W—H2WB	113.1
C4—C5—H5A	118.1		
			
O3W—Cd1—N1—C5	−12.4 (3)	O3W—Cd1—O2—C12	5.4 (2)
O4—Cd1—N1—C5	122.9 (3)	O4—Cd1—O2—C12	−172.26 (19)
N2^i^—Cd1—N1—C5	−88.0 (11)	N2^i^—Cd1—O2—C12	−84.20 (19)
O5—Cd1—N1—C5	70.6 (2)	N1—Cd1—O2—C12	92.11 (19)
O3—Cd1—N1—C5	−97.2 (3)	O5—Cd1—O2—C12	161.38 (17)
O2—Cd1—N1—C5	−150.6 (3)	O3—Cd1—O2—C12	−2.13 (17)
C14—Cd1—N1—C5	96.7 (3)	C14—Cd1—O2—C12	177.63 (18)
O3W—Cd1—N1—C1	168.4 (3)	O3W—Cd1—O3—C12	−172.87 (19)
O4—Cd1—N1—C1	−56.3 (3)	O4—Cd1—O3—C12	17.4 (2)
N2^i^—Cd1—N1—C1	92.7 (11)	N2^i^—Cd1—O3—C12	100.03 (19)
O5—Cd1—N1—C1	−108.6 (3)	N1—Cd1—O3—C12	−80.64 (19)
O3—Cd1—N1—C1	83.6 (3)	O5—Cd1—O3—C12	−131.0 (3)
O2—Cd1—N1—C1	30.1 (3)	O2—Cd1—O3—C12	2.10 (17)
C14—Cd1—N1—C1	−82.6 (3)	C14—Cd1—O3—C12	1.2 (4)
C5—N1—C1—C2	−0.4 (6)	O3W—Cd1—O4—C14	23.0 (2)
Cd1—N1—C1—C2	178.9 (4)	N2^i^—Cd1—O4—C14	106.73 (19)
N1—C1—C2—C3	1.8 (7)	N1—Cd1—O4—C14	−75.40 (19)
C1—C2—C3—C4	−1.5 (7)	O5—Cd1—O4—C14	−1.53 (16)
C2—C3—C4—C5	−0.1 (6)	O3—Cd1—O4—C14	−171.49 (16)
C2—C3—C4—C6	177.0 (4)	O2—Cd1—O4—C14	−159.18 (19)
C1—N1—C5—C4	−1.3 (5)	Cd1—O5—C14—O4	−2.7 (3)
Cd1—N1—C5—C4	179.4 (2)	Cd1—O5—C14—C15	175.8 (3)
C3—C4—C5—N1	1.6 (5)	Cd1—O4—C14—O5	2.8 (3)
C6—C4—C5—N1	−175.7 (3)	Cd1—O4—C14—C15	−175.8 (3)
C3—C4—C6—O1	−156.8 (4)	O3W—Cd1—C14—O5	19.8 (2)
C5—C4—C6—O1	20.3 (5)	O4—Cd1—C14—O5	−177.3 (3)
C3—C4—C6—C9	21.1 (5)	N2^i^—Cd1—C14—O5	108.46 (18)
C5—C4—C6—C9	−161.8 (3)	N1—Cd1—C14—O5	−71.88 (18)
C11—N2—C7—C8	1.5 (5)	O3—Cd1—C14—O5	−153.9 (3)
Cd1^ii^—N2—C7—C8	−170.5 (3)	O2—Cd1—C14—O5	−154.70 (17)
N2—C7—C8—C9	1.3 (5)	O3W—Cd1—C14—O4	−162.90 (17)
C7—C8—C9—C10	−3.2 (5)	N2^i^—Cd1—C14—O4	−74.26 (19)
C7—C8—C9—C6	176.7 (3)	N1—Cd1—C14—O4	105.40 (19)
O1—C6—C9—C8	33.5 (5)	O5—Cd1—C14—O4	177.3 (3)
C4—C6—C9—C8	−144.4 (3)	O3—Cd1—C14—O4	23.4 (4)
O1—C6—C9—C10	−146.6 (4)	O2—Cd1—C14—O4	22.6 (2)
C4—C6—C9—C10	35.5 (5)	O3W—Cd1—C14—C15	−89 (4)
C8—C9—C10—C11	2.5 (5)	O4—Cd1—C14—C15	74 (4)
C6—C9—C10—C11	−177.4 (3)	N2^i^—Cd1—C14—C15	−1 (4)
C7—N2—C11—C10	−2.2 (5)	N1—Cd1—C14—C15	179 (100)
Cd1^ii^—N2—C11—C10	169.5 (3)	O5—Cd1—C14—C15	−109 (4)
C9—C10—C11—N2	0.3 (5)	O3—Cd1—C14—C15	97 (4)
O3W—Cd1—O5—C14	−161.18 (19)	O2—Cd1—C14—C15	96 (4)
O4—Cd1—O5—C14	1.54 (17)	Cd1—O2—C12—O3	3.8 (3)
N2^i^—Cd1—O5—C14	−76.13 (19)	Cd1—O2—C12—C13	−175.3 (3)
N1—Cd1—O5—C14	105.42 (18)	Cd1—O3—C12—O2	−3.8 (3)
O3—Cd1—O5—C14	156.8 (2)	Cd1—O3—C12—C13	175.3 (3)
O2—Cd1—O5—C14	34.7 (2)		

Symmetry codes: (i) *x*−1, *y*+1, *z*; (ii) *x*+1, *y*−1, *z*.

### Hydrogen-bond geometry (Å, º)

**Table:**

*D*—H···*A*	*D*—H	H···*A*	*D*···*A*	*D*—H···*A*
O1*W*—H1*WA*···O4	0.89	2.06	2.773 (2)	136
O1*W*—H1*WB*···O2w	0.89	2.00	2.834 (3)	155
O2*W*—H2*WA*···O2	0.89	1.93	2.811 (4)	173
O2*W*—H2*WB*···O1w^iii^	0.89	1.95	2.803 (2)	162
O3*W*—H3*WA*···O5^iv^	0.89	1.80	2.679 (2)	172
O3*W*—H3*WB*···O3^v^	0.89	1.81	2.693 (3)	170

Symmetry codes: (iii) −*x*+1, −*y*+2, −*z*+1; (iv) −*x*+2, −*y*+1, −*z*+2; (v) −*x*+1, −*y*+1, −*z*+2.
